# Association of the *MTHFR* C677T polymorphism and bone mineral density in postmenopausal women: a meta-analysis

**DOI:** 10.1016/S1674-8301(10)60056-5

**Published:** 2010-11

**Authors:** Donghua Li, Jie Wu

**Affiliations:** Department of Obstetrics and Gynecology, the First Affiliated Hospital of Nanjing Medical University, Nanjing 210029, Jiangsu Province, China

**Keywords:** methylenetetrahydrofolate reductase, postmenopausal women, bone mineral density, meta-analysis

## Abstract

Osteoporosis is a condition characterized by low bone mineral density (BMD) and micro-architectural changes in the bone tissue. The risk of osteoporosis is partly determined by genetic factors. The role of C677T polymorphism of methylenetetrahydrofolate reductase (*MTHFR*) gene has been investigated in postmenopausal osteoporosis. However, the relationship between *MTHFR* polymorphism and BMD is still controversial. We carried out a meta-analysis of 5,833 subjects to evaluate the association of *MTHFR* and BMD in postmenopausal women. Databases of MEDLINE, Web of Science, Scopus and CNKI were retrieved for all publications relating to *MTHFR* polymorphism and BMD in postmenopausal women. Five eligible studies were selected for meta-analysis. All these articles studied the association of *MTHFR* polymorphism and BMD of the femoral neck and lumbar spine in postmenopausal women. Our analysis suggested that postmenopausal women with the TT genotype had lower femoral neck BMD than the women with the CC/CT genotype, and the weighted mean difference (WMD) was -0.01 g/cm^2^ [95% confidence interval (CI): (-0.01, -0.01), *P* < 0.01]. However, BMD of the lumbar spine of postmenopausal women with the TT genotype was not significantly different from that of women with the CC/CT genotype. In the random effects model, the WMD between the TT and TC/CC genotype was -0.01 g/cm^2^ [95% CI: (-0.04, 0.01), *P* = 0.32]. The C677T polymorphism of the *MTHFR* gene is associated with BMD of the femoral neck in postmenopausal women. Women with the TT genotype of the *MTHFR* gene have lower BMD, suggesting that the TT genotype may be a risk factor for postmenopausal osteoporosis.

## INTRODUCTION

Osteoporosis is a common metabolic bone disorder characterized by reduced bone mass, increased skeletal fragility and micro-architectural deterioration, and, as a consequence, increased bone fracture[1]. According to statistics, 30% of women and 12% of men may suffer from osteoporosis at some point during their lifetime, and osteoporosis is becoming a major economic burden on the society and families[2]. In the United States alone, there are over 1.5 million fractures each year, including 280,000 hip fractures and 500,000 vertebral fractures[3]. According to one report,[4] the prevalence of osteoporosis is 40%-50% in women aged over 60 y, and osteoporotic fracture may occur in 30%-50% of this group. This remains a significant and growing public health concern. Moreover, postmenopausal osteoporosis also increased the risk of fractures[5],[6], especially hip and spine fractures, which are associated with high morbidity and mortality in this population[7],[8]. Because bone fracture prevention is the primary aim of osteoporosis treatment, an assessment of bone strength is absolutely required to determine fracture risk in patient. According to the definition of osteoporosis proposed by the National Institutes of Health[1], bone strength is determined by the bone mass (bone mineral density) and bone quality. Among these two components, bone mineral density (BMD) is the primary factor affecting susceptibility to osteoporotic fracture[9],[10]. Presently, BMD of postmenopausal women is still the gold standard for the assessment of fracture risk[11],[12].

The risk factors of osteoporosis are partly determined by genetic factors[13]. Identifying genes associated with BMD would be useful for predicting bone mass and clarifying possible mechanisms of osteoporosis[14]. Identifying persons at increased risk for osteoporotic fractures is thus very important since a variety of preventive treatments are now available. Genetic markers could be useful since they could make screening program easier and more cost effective by improving fracture prediction beyond that provided by BMD measurement alone. With improved fracture prediction, intervention may become feasible earlier. In studies of twins, it has been reported that approximately 75% of the inter-individual variance of bone mass is determined by genetic factors[15]. Multiple genes related to bone metabolism or structure, such as the vitamin D receptor gene, the collagen type IA 1 gene and the estrogen receptor gene, have been studied in this context. Methylenetetrahydrofolate reductase (*MTHFR*) gene polymorphism has been identified as a candidate gene for osteoporosis in postmenopausal women since Miyao *et al.*[16] studied the association of *MTHFR* polymorphism with BMD in postmenopausal Japanese women. The *MTHFR* gene lies within the 1p36 region on chromosome 1[17], and it catalyzes the conversion of 5, 10 methylenetetrahydrofolate to 5-methyltetrahydrofolate. Therefore, severe deficiency of *MTHFR* can cause hyperhomocysteinemia due to the lack of 5-methyltetrahydrofolate[18]. Two recent population-based studies from the Netherlands and Framingham, USA, have shown an association between increased concentrations of plasma total homocysteine (tHcy), and the risk of osteoporotic fracture in both male and female subjects[14],[19]. Condon 677 (C677T) has been identified as a functional polymorphism in *MTHFR*. The T-allele variant (valine type) has lower enzyme activity than the wild type (C-allele or alanine type), resulting in a slightly elevated homocysteine level, and this has been recently recognized as a risk factor for fracture. The TT genotype is associated with slightly increased homocysteine levels with variable effects on BMD and fracture risk. Abrahamsen *et al.*[20] have published data from the Danish Osteoporosis Prevention Study (DOPS) showing that healthy early post-menopausal Danish women with the TT genotype have 0.1–0.3 SD reduced BMD at most measurement sites and that one in nine fractures in this age group could be attributed to this genetic polymorphism alone. Some studies suggested that the C677T polymorphism in the *MTHFR* gene was associated with BMD of postmenopausal women[21]-[25]. In addition, several reports showed that *MTHFR* polymorphism was associated with an increasing risk of postmenopausal osteoporotic fractures[26]-[29]. However, the relationship between the *MTHFR* polymorphism and postmenopausal BMD is still controversial. In order to elucidate the correlation, we collected all articles published to date and performed a meta-analysis of the relationship between *MTHFR* gene polymorphism and BMD in postmenopausal women.

## MATERIALS AND METHODS

### Search strategy

The MEDLINE (1950-2010), Web of Science (1955-2010), Scopus (1960-2010) and China National Knowledge Infrastructure (CNKI, 1994-2010) were searched in a systematic and comprehensive manner for all previous studies on the *MTHFR* C677T polymorphism and BMD in postmenopausal women. The search used the following keywords: methylenetetrahydrofolate reductase, postmenopausal, bone mineral density. If abstracts were considered relevant, full-text articles were obtained and examined. The references of all computer-identified publications were searched for additional studies, and the MEDLINE related articles were used to search for potentially relevant articles. Review articles and references of other identified studies were hand-searched to find additional eligible studies. Articles published in all languages were selected if they met all of the following criteria: (1) study included postmenopausal women with femoral neck and/or lumbar spine BMD; (2) study stratified by *MTHFR* C677T genotypic class in TT and CC/CT genotype or in TT, CC and CT genotype; (3) the BMD of each participant was measured by dual-energy X-ray absorptiometry; (4) those that retrieved studies of review articles were excluded.

### Data extraction

Following the meta-analysis of observational studies in epidemiology (MOOSE) guidelines for reporting meta-analysis of observational studies[30], we extracted the following data from eligible studies: author's name, region/country where the study was conducted, year of publication, number of subjects, mean age or age range of all included subjects, how the subjects stratified by *MTHFR* C677T genotypic class, the number of cases in each group and the BMD value of the femoral neck and lumbar spine. All data were independently abstracted in duplicate by two researchers. If the researchers disagreed, a final result was reached by discussion. For data not provided in table form or in the main text, the required information was obtained by contacting corresponding authors when possible.

### Statistical analysis

Summary statistics were estimated in the Rev Man 4.2 software (The Nordic Cochrane Center, Rigshospitalet). As the value of BMD is continuous data, we used the weight mean difference (WMD) as the combined study effect size estimates and summary statistics with 95% confidence intervals (CI). The WMD was used as the common output measurement to assess the quantitative data, such as height, blood pressure, and biochemical indicators. It was estimated using fixed or random (in case of heterogeneity) effect models with RevMan 4.2 software. The sample size, mean value and standard deviations of the main outcome measures were extracted from the studies. A χ^2^ test was used to examine the included studies for statistical evidence of heterogeneity, and the degree of heterogeneity was assessed with the I2 statistic. We applied the fixed-effects model in case of no heterogeneity (*P* > 0.05). Otherwise, the random-effects model was used (*P* < 0.05). Results with a *P* value less than 0.05 were considered statistically significant.

## RESULTS

According to the search criteria set, there were fifteen published articles identified above for potential inclusion with full-text versions obtained for *MTHFR* and postmenopausal BMD. Two articles, by Macdonald HM *et al*.[31] and Nissen N *et al*.[32], were excluded because the subjects were not suitable for our meta-analysis as some of them were pre/peri-menopausal women. Three articles[16],[33],[34] were excluded too, because they were not stratified by *MTHFR* C677T genotypic class in TT and CC/CT genotype. Another paper by Abrahamsen B *et al*.[20] was also excluded because some of the subjects underwent hormone replacement therapy. In addition, four articles, written by Hong X *et al*.[2], Villadsen MM *et al*.[28], Baines M *et al*[35]. and *Jørgensen HL* *et al*.[36], respectively, were also excluded because the data can not be found in the tables or the main texts. Additionally we have tried to get in touch with the corresponding authors as possible as we can, but the required information was not obtained. Therefore, ten papers were excluded among fifteen identified, thus yielding five studies. The five studies[21]-[25] on the association between the *MTHFR* C677T polymorphism and BMD of the femoral neck in postmenopausal women involved 542 subjects with the TT genotype and 4,855 subjects with the CC/CT genotype, and the lumbar spine BMD data included 607 subjects with the TT genotype and 5,226 subjects with the CC/CT genotype, and detailed characteristics of each study are described in ***Table 1*** and ***Table 2***.

**Table 1:** Characteristics of included studies of the *MTHFR* genotype and BMD of the femoral neck

Study	Year	Country	Age (y)	TT genotype		CC/CT genotype		WMD	95%CI
				*n* (%)	BMD (g/cm^2^)	*n* (%)	BMD (g/cm^2^)		
Agueda L[21]	2010	Spain	57.6±8.2	79(15.55)	0.67 ± 0.01	429(84.45)	0.68±0.01	-0.01	(-0.01, -0.01)
Yazdanpanah N[22]	2008	Netherlands	66.4±8.2	296(11.00)	0.82 ± 0.13	2396(89.00)	0.84±0.14	-0.02	(-0.04, 0.00)
Golbahar J[23]	2004	Iran	60.8±6.8	6(2.21)	0.73 ± 0.08	265(97.79)	0.77±0.14	-0.04	(-0.11, 0.03)
Li M[24]	2004	China	57.3(55-59)	8(4.49)	0.66 ± 0.04	170(95.51)	0.68±0.10	-0.02	(-0.05, 0.01)
Abrahamsen B[25]	2003	Denmark	50.7±2.8	153(8.85)	0.78 ± 0.11	1595(91.25)	0.80±0.12	-0.02	(-0.04, 0.00)
Total				542(10.04)		4855(89.96)		-0.01	(-0.01, -0.01)

BMD: Bone mineral density; MTHFR: Methylenetetrahydrofolate reductase; WMD: Weighted mean difference.

**Table 2:** Characteristics of included studies of the *MTHFR* genotype and BMD of the lumbar spine

Study	Year	Country	Age (y)	TT genotype		CC/CT genotype		WMD	95%CI
				*n* (%)	BMD (g/cm^2^)	*n* (%)	BMD (g/cm^2^)		
Agueda L[22]	2010	Spain	55.5 ± 8.7	144(15.25)	0.86 ± 0.01	800(84.75)	0.85 ±0.01	0.01	( 0.01, 0.01)
Yazdanpanah N[23]	2008	Netherlands	66.4 ± 8.2	296(11.00)	1.03 ± 0.18	2396(89.00)	1.04±0.18	-0.01	(-0.03, 0.01)
Golbahar J[24]	2004	Iran	60.8 ± 6.8	6(2.21)	0.94 ± 0.17	265(97.79)	0.91± 0.13	0.03	(-0.11, 0.17)
Li M[25]	2004	China	57.3(55-59)	8(4.49)	0.82 ± 0.06	170(95.51)	0.86±0.12	-0.04	(-0.09, 0.01)
Abrahamsen B[21]	2003	Denmark	50.7 ± 2.8	153(8.85)	1.00 ± 0.13	1595(91.25)	1.03 ± 0.14	-0.03	(-0.05, -0.01)
Total				607(10.41)		5226(89.59)		-0.01	(-0.04, 0.01)

BMD: Bone mineral density; MTHFR: Methylenetetrahydrofolate reductase; WMD: Weighted mean difference.

**Fig. 1:**
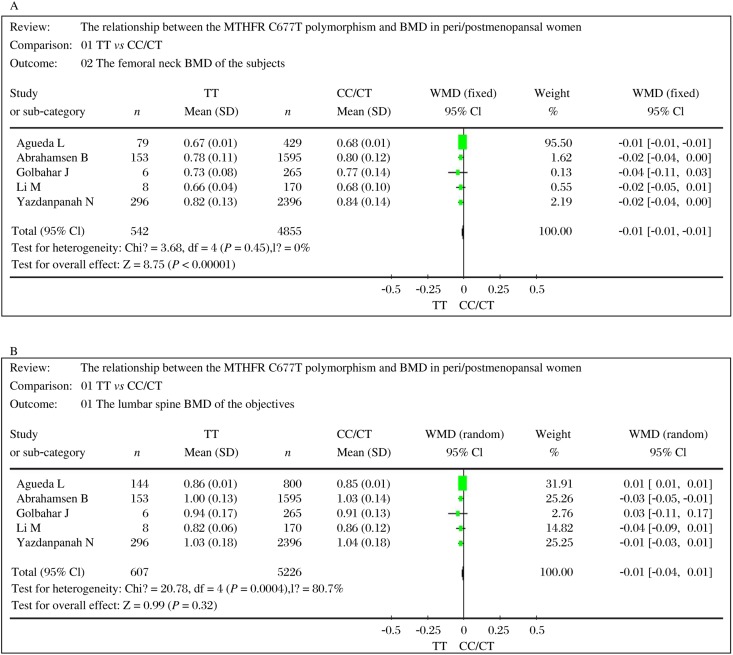
Individual and pooled weighted mean difference estimates and their 95% confidence intervals (CI) for femoral neck and lumbar spine bone mineral density (BMD) in postmenopausal women. The size of the square is proportional to the percent weight of each study in the fixed and random effect meta-analysis. Horizontal line represents the 95% CI. The summary pooled WMD and its 95%CI are indicated by the shaded diamond. A: femoral neck BMD in postmenopausal women. B: lumbar spine BMD in postmenopausal women.

After performing the tests for heterogeneity for the *MTHFR* C677T polymorphism and BMD of the femoral neck in postmenopausal women, we decided to use a fixed-effect model to obtain a summary statistic as the tests were not statistically significant (χ^2^ = 3.68, df = 4, *P* = 0.45 > 0.05). However we used a random-effect model in lumbar spine BMD as the tests were statistically significant (χ^2^ = 20.78, df = 4, *P* = 0.0004 < 0.05). In this meta-analysis, a statistically significant difference in the *MTHFR* C677T polymorphism and BMD of the femoral neck was observed in postmenopausal women and the WMD between the TT and TC/CC genotype was -0.01 g/cm^2^(95% CI: -0.01 to -0.01, *P* < 0.00001). But in BMD of the lumbar spine, the difference was not statistically significant and the WMD was-0.01 g/cm^2^ (95% CI: -0.04 to 0.01, *P* = 0.32), as shown in ***Fig. 1A*** and ***Fig. 1B***.

## DISCUSSION

As the association between *MTHFR* polymorphism and BMD in postmenopausal women is controversial, we searched the published articles and ideneifred some studies on the association between the *MTHFR* C677T polymorphism and BMD. However, ten studies are not suitable for this meta-analysis. In our data, five studies about the association between *MTHFR* polymorphism and postmenopausal BMD of the femoral neck and the lumbar spine were analyzed. Our findings suggested that postmenopausal women with the TT genotype had lower femoral neck BMD than that of women with the CC/CT genotype [WMD: -0.01 g/cm^2^, 95% CI: (-0.01, -0.01), *P* < 0.001]. But the lumbar spine BMD of postmenopausal women with the TT genotype had no significant difference compared with the CC/CT genotype [WMD: -0.01 g/cm^2^, 95% CI: (-0.04, 0.01), *P* = 0.32]. Our result was in accordance with the study by Riancho *et al*.[37] on the BMD of the femoral neck but inconsistent with the BMD of the lumbar spine in women.

Abrahamsen *et al*.[25] performed a prospective study of risk factors for osteoporosis in 1,748 healthy postmenopausal Danish women. They found that postmenopausal BMD at the femoral neck and lumbar spine of the subjects was significantly different between subjects with the TT genotype and CC/CT genotype, which suggestes that the less prevalent genotype (TT, 8.7% of the population) of the *MTHFR* gene was associated with lower postmenopausal BMD at the femoral neck (*P* < 0.05), lumbar spine (*P* < 0.05) and total hip (*P* < 0.01).

The analysis by Yazdanpanah *et al*.[22] showed that no association between the *MTHFR* C677T polymorphism and baseline BMD at femoral neck (*P* = 0.19) and lumbar spine (*P* = 0.26) in postmenopausal women. But there was a trend that the women with *MTHFR* TT genotype had lower BMD than that of the CC/CT genotype women, the result was not significantly different.

Golbahar *et al*.[23] reported that BMD was decreased in the postmenopausal women with the TT genotype, but BMD among the women with the CC, CT and TT genotypes was not statistically different at both the femoral neck (*r* = -0.01, *P* = 0.81) and lumbar spine (*r* = -0.04, *P* = 0.51). However, the sample size in the TT genotype group was very small. Agueda *et al*.[21] performed association analysis of three functional polymorphisms in a cohort of 944 postmenopausal Spanish women. They found that no significant association was observed between the studied polymorphisms and BMD of the lumbar spine or femoral neck. Li *et al*.[24] also reported that there was no significant difference in the *MTHFR* C677T polymorphism and BMD in 178 Chinese postmenopausal women.

Take together, five articles in our study showed similar results, which were in contrast to what was reported by Abrahamsen *et al*.[25] who observed a significant association between the *MTHFR* polymorphism and BMD in postmenopausal women. Our result, inconsistent with four of the five, was that the C677T polymorphism of the *MTHFR* gene is significantly associated with BMD of the femoral neck in postmenopausal women. There was a statistically significant difference in BMD of the femoral neck between the TT genotype and CC/CT genotype. Small sample size was thought to be one of the causes of this conflict. Another reason could be ascribed to the small proportion of the TT genotype analyzed by Golbahar[23] and Li[24]. There were only 6 (2.21%) and 8 (4.49%) postmenopausal women, respectively, in the group of the TT genotype. However the distribution of the TT genotype in normal Chinese and Iranian women was 14.10% and 6.4% in other researches[38],[39]. However, there was no statistically significant difference of BMD in the lumbar spine between the TT and CC/CT genotype. The inconsistency of the analysis could be influenced by different measurement apparati, sites and different grouping methods[40].

Although the relatively small number of studies limited the power of our meta-analysis, the results clearly suggested that the C677T polymorphism of the *MTHFR* gene was associated with BMD of the femoral neck and lumbar spine in postmenopausal women. The women with the TT genotype of *MTHFR* have lower BMD, which suggests that the TT genotype could be a risk factor for postmenopausal osteoporosis. More of futrue studies with a larger sample size are needed.
